# Construction and validation of podcast for teen sexual and reproductive health education*

**DOI:** 10.1590/1518-8345.6263.3705

**Published:** 2022-10-03

**Authors:** Paloma Loiola Leite, Francisco Ayslan Ferreira Torres, Leonarda Marques Pereira, Adriana de Moraes Bezerra, Lucas Dias Soares Machado, Maria Rocineide Ferreira da Silva

**Affiliations:** 1 Universidade Regional do Cariri, Departamento de Enfermagem, Iguatu, CE, Brasil.; 2 Universidade Estadual do Ceará, Fortaleza, CE, Brasil.; 3 Fundo Estadual de Combate à Pobreza - FECOP Extensão, Universidade Regional do Cariri, Brasil.

**Keywords:** Adolescent Health, Adolescent Behavior, Health Education, Sex Education, Educational Technology, Validation Study

## Abstract

**Objective:**

to construe and validate a podcast for teen sexual and reproductive health education.

**Method:**

a methodological study was conducted based on Freire’s perspective. The podcast was construed based on the Knowledge about Sexuality Questionnaire applied to 60 adolescents and an integrative literature review. Eleven experts conducted the validation process. Internal consistency was evaluated using Item-level Content Validity Index ≥0.78 and Cronbach’s alpha ≥0.700.

**Results:**

four podcast episodes were produced with the adolescents’ participation using the radio play format, addressing sex and sexuality, contraceptive methods, human immunodeficiency virus (HIV) and Acquired Immunodeficiency Syndrome (AIDS) and other sexually transmitted infections. The podcast episodes last between 8 and 11 minutes and was validated with Item-level Content Validity Index = 0.87 and Cronbach’s alpha = 0.951.

**Conclusion:**

the podcast was validated for teen sexual and reproductive health education and constitutes a tool for health professional practices, particularly nurses, as well as autonomous use by adolescents.

## Highlights

(1) Adolescents helped construct the technology.

(2) Podcast is an easy-to-access, low-cost educational resource.

(3) The radio play format emulates the target audience’s style and language.

(4) The podcast format is little used by nursing, especially in health education.

(5) The unpublished and judge-validated podcast is available for free.

## Introduction

Sexual and reproductive health education is permeated by censorship and restrictions based on prejudices, taboos and power relations. As this dimension concerns the life cycle, these limitations are even more noticeable when dealing with adolescents, with actions promoting individual accountability, shaming and behavior changes vertically guided by non-inclusive public policies^1^.

Teenagers, especially those living in developing countries, face additional challenges in terms of sexual and reproductive health, given the sex-related misconceptions, adult negligence and/or inattention, social, cultural and access barriers^2^.

Adolescence is marked by growth spurts, manifestation of secondary sexual characters, and cognitive transformations that corroborate the appreciation of new intrapersonal, interpersonal, and environmental bonds^3^. Hence, sexuality finds a locus of greater development and self-perception in this period.

Discussing sexual health involves discussing the act itself, including practices and experiences related to satisfaction, pleasure, affection, feelings and health. Sexual experiences, although presenting similar traits between individuals, are of unique development, modified according to the social and cultural context in which the adolescent lives, and may represent risks to health, quality of life and be a factor of vulnerability^2^.

In our understanding, therefore, we must advance the debate about the full exercise of sexuality in adolescence, providing subsidies to promote adolescent health in the dimensions of pleasure, intimacy and fulfillment. We seek to strength adolescent autonomy and empowerment, overcoming normative-preventive discourses that envision sexual health as a risk^4^.

Educational technologies shows potential in the education process to promote adolescent health, as it allows audience participation and the approach of different topics, resulting in inclusion and adequacy to health needs^5^.

Among the technologies used in health-related fields, the podcast has been gaining relevance in educational processes^6^ ^-^ ^7^. It consists of an online audio resource, accessed via computer, mobile phone and audio players, capable of gathering various information, such as lectures, interviews and commentaries^6^.

Its novel character lies in the flexibility of its modes of reproduction and sharing; autonomy in its use in a place and time relevant to the user; and in the dissemination of knowledge that overcomes geographical barriers, such as that imposed by the global COVID-19 pandemic^6^ ^,^ ^8^.

Under health promotion, health education proposals consolidated on the recognition of needs, health planning, teamwork, popular education and participation, represent advances consistent with assertive professional performance before the new modes of health production in the territories^9^.

In this context, the podcast becomes a relevant tool to facilitate teen access to information about sexual and reproductive health, free of inter-relational constraints and incentivizing self-care. It is also a powerful tool for professional health practice as an educational resource.

Hence, this study sought to create and validate a podcast for teen sexual and reproductive health education.

## Method

### Study design

This is a methodological study^10^ based on Freire’s perspective^11^ for the construction of an educational technology, divided into four stages: theoretical framework, identification of themes, elaboration of educational technology and validation of the material (Figure 1).

**Figure 1:**
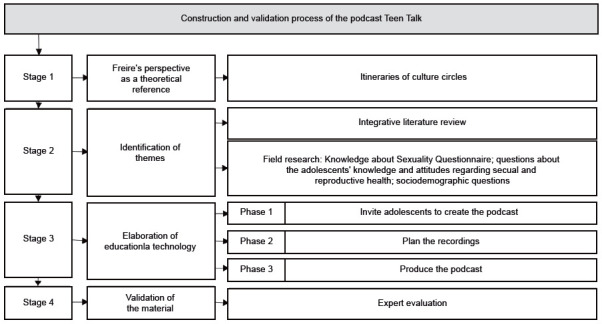
Algorithm for constructing and validating the podcast *Coisa de Adolescente* (Teen Talk). Iguatu, Ceará, Brazil, 2021

### Stage 1 - Freire’s perspective as a theoretical reference

Paulo Freire’s work on critical pedagogy, adopted here as a theoretical reference, argues that individuals apprehend their study subject through a dialectical interaction with reality. It consists, therefore, in an ethical, liberating and transformative education which relates with the different ways one see and experience the world, seeking to consciously transform it^11^.

We sought to mobilize popular education, participation and dialogue as essential elements to construct the podcast aiming at meaningful learning, drawing on the knowledge, experiences and community of the target public^11^.

Among Freire’s propals, we adopted his itinerary of the culture circle to guide the construction of the podcast, encompassing the stages of thematic research, to unveil the teen’s knowledge, worldview, previous experiences and vocabulary; coding and decoding, to investigate and debate the topics listed for later scripting; and critical unveiling, in which the proposed episode scripts are seen as products of a participative and dialectical process^12^.

### Stage 2 - Identification of themes

To bring together the theoretical framework, the relevant teen-related health education themes and the use of podcasts as educational technology, we conducted an integrative literature review. This was based on six steps: definition of research question, search and selection of primary studies, data extraction, critical evaluation of primary studies, summary of results and presentation of review^13^.

The research question, “What themes and educational technologies have been used to promote adolescent health?”, was formulated following the PICO strategy (Patient, Intervention, Comparison, Outcome)^5^.

Search and selection of primary studies was conducted on the Coordination of Higher Education and Graduate Training (CAPES), Latin American and Caribbean Health Sciences Literature (LILACS), Medical Literature Analysis and Retrieval System Online (MEDLINE) via PubMed, Cumulative Index to Nursing and Allied Health Literature (CINAHL), and Scientific Electronic Library Online (SciELO) databases using advanced search^5^.

We used the following search strategies: 1) Educational Technology AND Adolescent AND Health Promotion; 2) Educational Technology AND Adolescent; 3) Educational Technology AND Health Promotion; e 4) (“Educational Technology” OR “Instructional Technology” OR “multimedia”) AND (“Health Promotion” OR “Promotion of Health” OR “Promotional Itens”) AND (“Adolescents” OR “Adolescence” OR “Teens” OR “Teenagers” OR “Youth”)^5^.

Data were extracted according to an instrument constructed by the authors focused on pinpointing the prevalent themes and the technologies used to promote teen health. Methodological quality was evaluated using the Critical Appraisal Skills Program (CASP). Results were summarized and presented as texts^5^.

To understand the sexual and reproductive health education needs, complement the bibliographic survey and ensure teen participation, we applied the Knowledge about Sexuality Questionnaire (QCS)^14^.

### Stage 3 - Elaboration of educational technology

This stage focused on constructing the podcast and consisted of three phases: inviting adolescents to collaborate in the project, planning the recordings and producing the podcast. We adopted the radio play format, which involves theater, literature and music, to produce a dramatization outside the simple narration, creatively discussing everyday themes.

After listing the themes to be discussed, we planned the critical appropriation, wrote the scripts and recorded the episodes.

Based on Freire’s culture circles and concepts of popular education, participation and dialogue, the construction emerged from the interaction between adolescents interested in participating in the process and nursing undergraduates, advised by the proposing researcher.

### Stage 4 - Validation of the material

In this stage, the podcast was validated by experts, who were invited to suggest exclusions, additions or changes to parts or the whole technology. For validation, experts closely examine the technology construed based on established scores, quantifying their agreement with content validity.

### Study setting

The study was conducted at a state vocational school, which offers vocational high school education to adolescents, providing technical training in Business Administration, Computing, and Nursing. The institution was chosen for convenience, considering the partnership to execute the activities of the extension project and develop a school radio, involving the students in the production, editing, and dissemination of audio content in the school environment, such as the podcast.

### Period

The study was conducted between March 2020 and June 2021.

### Population, selection criteria and sample

The study population consisted of 440 adolescents enrolled at the chosen school. Due to the sanitary and epidemiological conditions of the period, resulting from the COVID-19 pandemic, about 30% (n=132) of the students were regularly attending the school activities via remote classes and/or classroom activities. Sampling was performed using G*Power 3.1.9.7, setting a 0.05 sampling error, 0.95 confidence level and 0.50 estimated effect. We obtained a sample of 54 adolescents, and the final sample included 60 adolescents. A faculty member and ten nursing students also participated in conducting the study phases, as well as eleven experts.

Selection criteria consisted of adolescents between 10 and 19 years of age - delimited according to the Ministry of Health - enrolled regularly and attending school activities. Adolescents who failed to return the data collection instrument on the previously agreed upon deadline were excluded.

Experts on health education and/or public/collective health, digital media, educational technologies or audio, were assigned a score from five to eight points as selection criterion. Those who achieved the minimum score of five points, corresponding to thesis or dissertation on the theme(s) (2 points), undergraduate or specialization monograph on the theme(s) (1 point), participation in research groups/projects involving the theme(s) (1 point), teaching experience (1 point), practical experience (1 point), advisor for research on the theme(s) (1 point), and participation in evaluation boards for papers on the theme(s) (1 point)^15^, were invited to participate in the study.

An invitation was sent to the e-mail registered on the Lattes platform, available on the National Council for Scientific and Technological Development (CNPq) website, and through the snowball technique, in which one participant indicates a new participant according to their background and suitability to the study goals.

Validation was conducted by means of an instrument previously used to validate a podcast for health education on leprosy, consisting of items related to content (8 items), functionality (6 items), appearance (9 items) and sound environment (7 items)^16^. Experts attributed agreement to each item based on a 4-point Likert scale: 1 - irrelevant, 2 - little relevant, 3 - relevant, 4 - highly relevant.

### Study variables

The online questionnaire used for data collection, applied via Google Forms, included sociodemographic questions for the adolescents (gender, age in complete years, ethinicity/color, religion, and marital status) and for the experts (gender, age, degree, title and teaching field, research, care, and/or management).

### Instrument used for data collection

To identify the adolescents’ knowledge needs, we applied the Knowledge about Sexuality Questionnaire (QCS). This instrument consists of 25 statements with dichotomous answers (true/false), divided into six dimensions: first sexual intercourse and sexual concerns, sexuality and sexual pleasure, contraception and safe sex practices, pregnancy prevention, Sexually Transmitted Infections (STI) and Human Immunodeficiency Virus/Acquired Immune Deficiency Syndrome (HIV/AIDS), and sexual and reproductive health counseling and care^14^.

We also formulated additional questions about their knowledge and attitudes regarding sexual and reproductive health, such as: knowledge about STIs, how to protect oneself, use of condoms in sexual relations, situations in which they do not use condoms, known contraceptive methods, how to obtain condoms and the main source of information on sexual and reproductive health.

### Data collection

To survey the teenagers’ knowledge needs, data collection followed the agreement by the institution’s coordination: presentation of the proposal and material to the teachers and agreement as to its forwarding to the students via access link to the questionnaire and explanatory video produced by the nursing undergraduates presenting the instrument and explaining the consent form to be signed by a person responsible and the assent and proper completion of the instrument. Physical copies of the instrument were made available to the adolescents who requested it.

Concerning the podcast, the teenagers were invited to participate in the construction of the technology via Whatsapp^®^. During the second stage, we explained the objective and the operational process to create the podcast, which included planning the recordings, defining the genre podcast and its use in educational activities, and explaining what a radio play is.

Each episode was scripted and recorded in three meetings, conducted according to the itineraries of culture circles. In these moments, the adolescents discussed the themes, prepared the script and recorded the episode. These were facilitated by a researcher, a doctoral student with previous experience working with adolescents and health education, with the help of nursing students.

Such discussions, used to structure the scripts, involved a collective production in a conversation circle among the participants guided by questions like “tell me about,” such as tell me about HIV and AIDS. From then on, we sought to verify the participants’ knowledge, terms used to discuss the theme, and aspects which demonstrated uncertainty or erroneous knowledge. Further knowledge was provided by the researcher, clarifying doubts. For example, faced with the theme Sexually Transmitted Infections, it was necessary to know the main infections that affect adolescents, their forms of transmission, treatment options, forms of prevention, among others.

The meetings to discuss the theme and write the script lasted an average of two and a half hours, while the recording lasted about an hour.

The third stage comprised the pre-production, production, and post-production of the podcast. In pre-production the adolescents defined the characters and their actors and wrote the technical scripts, which were then appreciated by the coordinator, forwarding suggestions for adjustments as needed. In production, the radio play was recorded via Google Meet, respecting social distancing. Next, the audio from the video was extracted and sent to post-production, involving editing, inclusion of soundtrack and effects, mixing, and mastering in Cantasia Studio 9 software version 2020.012.

The educational technology was then made available to the experts for evaluation and validation, along with the evaluative instrument, invitation letter, and Informed Consent Form (ICF), assuring their desire to participate in the study. Three rounds of invitation/reminder for participation were sent at 15-day intervals.

### Data processing and analysis

The collected data were entered in a Microsoft Office Excel 2016^®^ spreadsheet and processed via Statistical Package for the Social Sciences (SPSS) version 20.

Sociodemographic variables, and the questions about adolescents’ knowledge and attitudes about sexual and reproductive health were investigated using descriptive statistics and presented as absolute and relative frequency, mean, and standard deviation (SD). For the Knowledge about Sexuality Questionnaire, we calculated the overall score and defined quintiles as levels of knowledge, according to correct answers to the items of each dimension: very unsatisfactory (0 to 20%), unsatisfactory (21 to 40%), average (41 to 60%), satisfactory (61 to 80%), and very satisfactory (80 to 100%).

Inter-expert agreement analysis was performed using Fleiss’ kappa test. We considered the variation from zero to one, where K<0.20 the agreement is very weak; 0.21≤K≤0.40 the agreement is weak; 0.41≤K≤0.60 the agreement is moderate; 0.61≤K≤0.80 the agreement is good; and ≥0.81 the agreement is very good^17^.

For data analysis, we calculated the Content Validity Index (CVI), which estimates inter-expert congruence on the evaluated aspects. The Item-level Content Validity Index (I-CVI) was calculated, corresponding to the number of experts who attributed 3 (relevant) or 4 (highly relevant) to a given item divided by the total number of experts, considering excellent the index greater than or equal to 0.78^18^. Cronbach’s alpha estimated the instrument’s internal consistency, with a desired value greater than or equal to 0.700^19^.

### Ethical aspects

The project was submitted to and approved by the Research Ethics Committee under opinion no. 4.205.242. Care was taken to follow national and international regulations for research involving human beings in all stages of the study. Adolescents signed an assent form, assuring their autonomy in contributing to the study, and an Informed Consent Form was sent for their parents and/or guardians. When filling out the online form, there was an option to confirm the agreement to participate in the study and a contact to send a scanned copy of the consent form duly filled out and signed.

## Results

Our results constructed and validated a podcast with four episodes on sexual and reproductive health aimed at teens, named Teen Talk.

The use of Circle of Culture itineraries contributed to ensure the participation of teens during episode theorization, scripting, and recording. In investigating our vocabulary universe, we sought to approximate the terms teens use to describe the relevant aspects of their sexual and reproductive health, such as “aunt flow” and “catch a disease,” referring to menstruation and sexually transmitted infections, respectively.

Then, from the themes and generating words we coded and decoded, we aimed to use situations, spaces, and preferences common to the realities of the participants in our episodes. Among these, we highlight meetings between teens in city squares, the predominance of educational activities and lectures in schools and basic health units, and teens’ contact with health information via digital media, such as live broadcasts on social networks.

To problematize the themes which emerged at our first meeting, we proposed to dialogically explore each specific theme. This problematization was essential to strengthen participants’ understanding of the singularities of the discussed theme and its representation in episode scripts.

A literature review to identify the themes and types of technologies used retrieved 19 studies, nine of which used tools to promote teens’ health, contemplating themes related to their sexual and reproductive health (47.36%). Moreover, nine studies used digital applications (47.36%). We also found printed technologies, such as games and comics, and action proposals, such as plays. We found no podcast for teens’ health education.

In total, 60 teens participated in our assessment of their knowledge and attitudes toward their sexual and reproductive health. They had a mean age of 17 years (SD=2.45) and were mostly single (83.3%), Brown (66.7%), and female (61.7%) Catholics (40%).

We found that teens especially used media/internet/television (43.3%) as their main information source. Most participants reported knowing HIV/AIDS STIs (90%), human papilloma virus (HPV) (75%), herpes (61.7%), and syphilis (61.7%). Their knowledge of granuloma inguinale and cancer was limited and 8.3% of participants showed no knowledge of any of the listed infections.

Regarding contraceptive methods, 85% of volunteers knew about condoms; 80%, hormonal methods; and 33.3%, natural methods, such as *coitus interruptus* and calendar-based ones. However, only 53.3% claimed to use condoms in their sexual relations. Among the situations participants claimed dispensed with contraception are fixed partners (46.7%), lacking condoms at the time (10%), and relations with someone with whom they were familiar (8.3%).

Their overall score at the General Sexual Knowledge Questionnaire was satisfactory, with 79.3% of correct answers. However, we found unsatisfactory levels of HIV testing and school sexuality counseling. Moreover, the items on sexual initiation and practices, relation of the influence of alcohol on perception and sexual behaviors, pregnancy due to sexual contact with unprotected penetration, and HIV transmission, showed participants’ regular knowledge. Thus, the themes listed were relevant to empower teens.

Surveying teens’ knowledge needs began the elaboration of educational technology. We recruited teens by the social network Whatsapp^®^ and extension students by the Teen Talk Extension Project. In total, five teens were willing to participate in all stages of the educational podcast construction.

It involved training participants on the podcast, its use, production stages, and a radio play. Overall, we chose four themes to produce four episodes: Sex and Sexuality; Contraceptive Methods; HIV/AIDS, and Other STIs. Students played all characters (whom they gave fictitious names) in the episodes. After construction, the groups sent their scripts to the teacher-advisor via e-mail to align the technical questions about the addressed information. Figure 1 summarizes the main characteristics of the recorded episodes.

**Figure 1:**
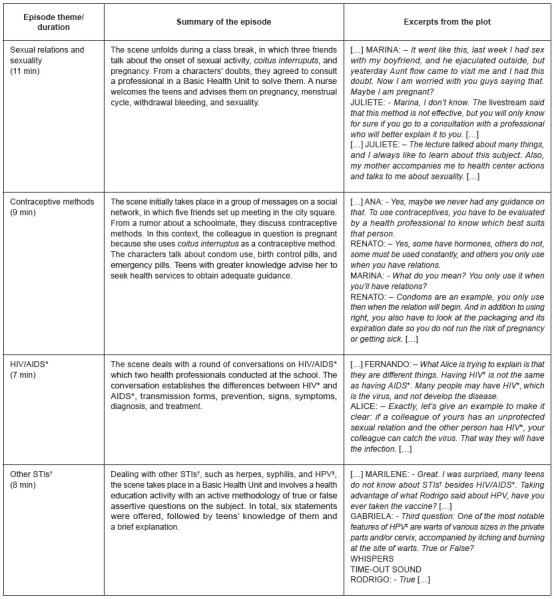
Summary of episodes of the Teen Talk podcast. Iguatu, CE, Brazil, 2021 *HIV/AIDS = Human immunodeficiency virus/acquired immunodeficiency syndrome; †STIs = Sexually transmitted infections; ‡HPV = Human papillomavirus

Before recording the material, we guided participants on recording techniques, tested the audio for each participant, and performed a first reading of the script, simulating the recording of the podcast so that each participant could adapt and incorporate their character, as well as adjust the offered language and content.

Episodes last between eight and 11 minutes to keep viewers’ attention, thus contributing to target its audience’s learning.

In total, 11 nursing specialists participated in the content validation stage, of which eight were women. All had experience in public/collective health and 63.3%, in health education/promotion. Moreover, 81.1% had experience in teaching, followed by experiences in care (36.3%) and research (18.1%). No specialist had management experience. All participants held masters’ degrees and their areas of study were health education/promotion (63.3%), public/collective health (81.8%), and digital media/education technology (36.3%).

The Fleiss kappa test showed specialists’ moderate reliability (K=0.58 [95% CI: 0.019-0.97]; z=2.938; p<0.05).

The educational podcast showed a 0.87 average I-CVI. Regarding item relevance, expressed by the I-CVI (which is equivalent to the number of specialists who agree or fully agree with a given item), we observed that its value was greater than 0.78 in most evaluated items, except for 4,1, 4.3, and 4.5 (Table 1).

We considered the internal consistency of the evaluated items as satisfactory, with a 0.951 Cronbach’s alpha for the used instrument.

**Table 1:** Item validity index and Cronbach’s alpha for content, functionality, appearance, and sound environment of the Teen Talk podcast. Iguatu, CE, Brazil, 2021

Evaluated items	I-CVI*	Cronbach’s Alpha
1. Content		
1.1 The content meets a possible health education situation.	1.00	0.954
1.2 The content is consistent with an educational health practice.	1.00	0.886
1.3 The content is relevant for health education.	1.00	0.954
1.4 The podcast shows cultural aspects of the population’s sexual and reproductive health reality.	0.82	0.795
1.5 The podcast can transmit educational health information to different audiences.	1.00	0.909
1.6 The podcast is enlightening about sexual and reproductive health.	1.00	0.886
1.7 The content is clear and objective.	1.00	0.886
1.8 The radio play aided content transmission.	1.00	0.931
2. Functionality		
2.1 The podcast is easily accessible.	1.00	0.909
2.2 Opening the podcast catches the attention of listeners and indicates the content of the material.	1.00	0.954
2.3 The language used is compatible with educational material.	1.00	0.886
2.4 The podcast suitably disseminates educational health material.	1.00	0.954
2.5 Podcast time is consistent.	1.00	0.909
2.6 The dramatization helped the audience to understand the content.	1.00	0.795
3. Appearance		
3.1 The title draws listeners’ attention.	1.00	0.840
3.2 The title is consistent with the content.	1.00	0.954
3.3 The duration of the podcast is satisfactory to provide knowledge about sexual and reproductive health.	1.00	0.840
3.4 The radio play motivates the audience to listen to the podcast.	1.00	0.886
3.5 The content and story shown encourage its audience to know/understand their sexual and reproductive health.	1.00	0.886
3.6 Its simple and clear scenes address sexual and reproductive health knowledge.	1.00	0.954
3.7 The narrative sequence is logic.	0.82	0.863
3.8 Listeners are encouraged to continue listening to the content until the end.	1.00	0.863
3.9 The formulation of dialogues is attractive and absorbing.	1.00	0.840
4. Sound Environment		
4.1 The scenarios and characters are attractive and identifiable by the used voice (locution) and sound effects.	0.73	0.704
4.2 Sound effects, locution type, and selected soundtracks facilitate the audience understanding the podcast.	0.82	0.795
4.3 It is easy to notice character changes by tone of the voice and locution type	0.73	0.772
4.4 It is easy to see environment changes by the used sound effects.	0.82	0.818
4.5 Characters are well described by their locution and by sound effects.	0.73	0.750
4.6 Track/sound effects helped the audience to understand the content.	1.00	0.886
4.7 The track/sound effects aided in setting the spaces.	0.82	0.840

*I-CVI = Item-level content validity index

**Figure 2:**
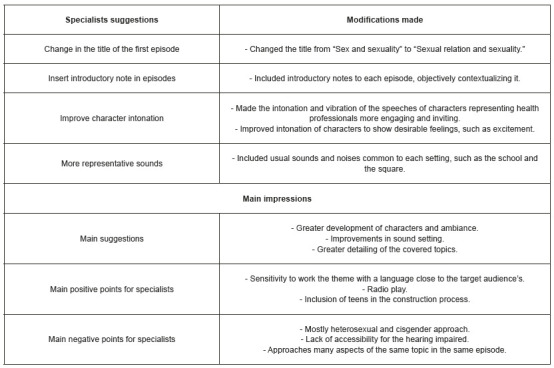
Suggestions from specialists and modifications made in the podcast. Iguatu, CE, Brazil, 2021

We elicited specialists’ recommendations, especially regarding the sound environment, a dimension with lower concordance indices, and performed the appropriate adjustments. Figure 2 describes specialists’ suggestions, modifications made, and evaluation impressions.

After this step, episodes were made available on various digital platforms (Deezer, Google Podcasts, Anchor, Amazon Music, Pocket Casts, Breaker, Spotify, Overcast, and Apple Podcasts) for appreciation and download, which the public can access free of charge and health professionals use in community education.

## Discussion

The podcast has become a popular asynchronous educational technology due to its ease of use, practicality, convenience, and repeatability, when compared to traditional teaching methods, and can offer much information since listeners only need a device which plays audio^20^. In the United States, for example, in 2019, 51% of the population over 12 years of age listened to podcasts^21^.

In this perspective, we developed the Teen Talk podcast to guide teens on topics relevant to their sexual and reproductive health in a way appropriate to their reality, ensuring their involvement in the whole process and providing continuous education, with access to content at any time and place.

The podcast is a communication tool which enables transmitters the possibility of disseminating educational materials in audio episodes on themes necessary to the community, increasing its efficiency by enabling users to perform other activities while enjoying their content^20^. With the insertion of Digital Information and Communication Technologies in teens and young people’s daily life, podcasts gain more and more space in learning environments, whether schools or health services.

Teens become the audience of health educational actions due to the vulnerabilities during this life phase, which favor a greater injury predisposition. We should stress that factors such as difficult family lives, contact with violence, religious and cultural conditions, low/lack of incentive to education, and curiosity toward risks, enable a greater possibility of acquiring STIs and/or unwanted pregnancies^22^.

A study conducted in public schools in Recife and Olinda (PE) indicates that health and education professionals use technological resources as differentials in educational actions on sexual and reproductive health since they bring educators closer to students, forming a tighter and more participatory support network on such a sensitive subject in this life phase. Innovating the approaches to sexual and reproductive health is relevant given teens’ daily technology use^23^.

Recording and listening to oneself and colleagues in developing the podcast better consolidates knowledge than reading materials on the same topic. Constructing this technology demands a greater commitment and analysis of the presented theme from involved individuals (in this case, teens and nursing students), expanding their participation in the production of knowledge and developing a more meaningful and participative learning^24^.

Nowadays, podcasts gain prominence by sharing knowledge via various possibilities and by offering educators and health professionals a better development of their teaching-learning process in educational activities. This is due to its encouraging characteristics, when inserted in didactic activities, since, in addition to the opportunity of presenting a theme in a contextualized way, it motivates debates on disciplinary or interdisciplinary issues in its listeners^25^.

Thus, the possibility of approaching subjects about their sexual and reproductive health via a radio play is opportune since teens can imagine and experience the transmitted scenes, finding contextual recognition and anchoring themselves to give meaning to the learning process. The motivation to listen to podcasts relates to the convergent interaction of humor resources and the theoretical approach^26^.

Talking about sexual and reproductive health requires a facilitating posture and a clear approach to bring daily situations from its audience since the closer the theme is to reality, the easier their understanding and feeling of safety to manifest themselves. Thus, when suggesting the use of a tool as a component in teens’ daily life, educators should be available to bond with this audience^27^.

A study using podcasts in medical education showed contributory concurrency: content producers used it to promulgate and disseminate knowledge, whereas listeners appropriated this content to empower themselves and better elaborate their doubts^26^, showing the contributing potential to both parties involved in the development and consumption of podcasts*.*

Because teens start their sexual life early, personal training via the insertion of this theme before they begin their sexual practices is important. So greater applicability chances in individuals’ lives enhanced them, educational actions must question concrete situations of life to enable critical thoughts which collaborate to reflect changes in reality^27^.

Sexual education, when developed before sexual debut, can contribute to raising awareness on safe sex. Thus, it is essential to provide spaces for reflection on sexual behaviors to reduce injury exposure^27^.

During this study, the Brazilian population was oriented to maintain social distancing, changing their routines, forms of organization, and social action due to the COVID-19 epidemiological and sanitary situation. These changes in ways of thinking and acting promoted adaptations in school and health educational actions. Thus, the need to enable digital learning tools, including health education, is urgently needed, and podcasts materialize themselves as a favorable tool for learning continuation since its language enables its audience to understand verbal and non-verbal interventions^20^.

The literature has shown that using this type of content in educational actions is an effective resource to build knowledge since they can be easily used on the go, integrated into personal space, time, and content^28^. However, including students in its production is as important as delivering ready material as they acquire the role of protagonists in the process, exercising their empowerment and autonomy and strengthening their confidence for future activities^24^.

This study found that the internal consistency of specialists’ response ranged from 0.704 to 0.954, showing that the information contained in the educational material is coherent and valid for use by its target audience. Validation studies, such as game studies on sexuality for teens, found similar values, whose Cronbach’s alphas ranged from 0.79^29^ to 0.88^30^.

Specialists’ suggestions and criticisms enabled an external unbiased look at the developed process, redirecting the proposal to better fit its objective.

The limitations of this study include prioritizing themes according to previous surveys of teens’ knowledge and the duration of the podcast episodes, which failed to thoroughly contemplate them. To do so, we would need to produce new episodes. We also highlight the restriction of access to content for people with hearing impairment, which we could solve by making our scripts available. Finally, the absence of studies with similar purposes in the literature reduces its comparative potential with other realities. We found that assessing the usability of this tool is relevant for teens.

This study advances nursing and health promotion by proposing the innovative use of podcasts to approach topics related to teens’ sexual and reproductive health, a tool built based on scientific literature and its target audience’s knowledge needs, ensuring their participation in its development. Our results contribute to the health education process by using podcasts in health promotion spaces, such as schools and basic health units, as well as teens’ self-instruction. Its innovative character is due to the scarce use of podcasts to educate in areas other than medicine and by the adequacy of its language to teens, expanding its usability and impact as an educational resource for learning.

## Conclusion

This study built and validated the educational Teen Talk podcast as an educational tool to promote teens’ sexual and reproductive health, developed in a participatory and shared way among all involved individuals. Its validation by a body of specialists reinforces its potential for thematically approaching teens’ sexual and reproductive health either in health professionals’ (such as nurses) spaces and actions or for teens’ autonomy.

Using podcasts as an educational resource has some advantages: low cost, easy access by smartphone, availability at any time and place, unlimited possibility of repetitions, accessible language, and suitability to the reality of the public to whom it is intended. The participatory use of audio in educational actions still preserves participants’ image since many teens refuse to participate due to shyness. Thus, educators find, in educational podcasts, a dynamic tool which simplifies content and facilitates health education teaching-learning.

## References

[1] Goldfarb ES, Lieberman LD. (2021). Three decades of research: the case for comprehensive sex education. J Adolesc Health.

[2] Gelehkolaee KS, Maasoumi R, Azzin AS, Nedjat S, Parto M, Hajiabadi IZ. (2021). Stakeholders’ perspectives of comprehensive sexuality education in Iranian male adolescences. Reprod Health.

[3] Silva AA, Gubert FA, VC Barbosa, Freitas RWJF, Vieira-Meyer APGF, Pinheiro MTM (2021). Health promotion actions in the School Health Program in Ceará: nursing contributions. Rev Bras Enferm.

[4] Silva CB, Motta MGC, Bellenzani R, Brum CN, Ribeiro AC (2022). Pregnancy in young people born with HIV: particularities in the contexts of exercising sexuality. Interface.

[5] Pereira LM, Leite PL, Torres FAF, Bezerra AM, Vieira CMA, Silva MRF (2021). Educational Technologies for adolescente health promotion: evidence from the literature. Rev Enferm UFPE on line.

[6] Ifedayo AE, Ziden AA, Ismail AB. (2021). Podcast acceptance for pedagogy: the levels and significant influences. Heliyon.

[7] Milligan KJ, Daulton RS, Clair ZT, Epperson MV, Holloway RM, Schlaudecker JD. (2021). Creation of a student-run medical education podcast: tutorial. JMIR Med Educ.

[8] Tarchichi TR, Szymusiak J. (2021). Continuing medical education in the time of social distancing: the case for expanding podcast usage for continuing education. J Contin Educ Health Prof.

[9] Machado LDSM, Xavier SPL, Maia ER, Vasconcelos MIO, Silva MRP, Machado MFAS (2021). Health promotion conceptions and expressions in the training process of the multi-professional residency. Texto Contexto Enferm.

[10] Polit DF, Beck CT. (2011). Essentials of Nursing Research: appraising evidence for nursing practice.

[11] Araujo BBM, Machado ACC, Rossi CS, Pacheco STA, Rodrigues BMRD (2018). Paulo Freire’s theoretical and methodological framework: contributions in the field of nursing. Rev Enferm UERJ.

[12] Souza JB, Barbosa MHPA, Schmitt HBB, Heidemann ITSB (2021). Paulo Freire’s culture circles: contributions to nursing research, teaching, and professional practice. Rev Bras Enferm.

[13] Mendes KDS, Silveira RCCP, Galvão CM. (2019). Use of the bibliographic reference manager in the selection of primary studies in integrative reviews. Texto Contexto Enferm.

[14] Carvalho CP, Pinheiro MRM, Gouveia JAP, Vilar DR. (2017). Conhecimentos sobre sexualidade: Construção e validação de um instrumento de avaliação para adolescentes em contexto escolar. Rev Portuguesa Educ.

[15] Fehring RJ. (1994). Symposium on validation models: the Fehring model. ln: Carrol-Johnson RM, Paquette M. Classification of Nursing Diagnoses.

[16] Muniz RAA (2017). Construção e validação de podcast com conteúdo educacional em saúde com participação ativa de acadêmicos de enfermagem.

[17] Altman DG (1990). Practical statistics for medical research.

[18] Polit DF, Beck CT, Owen SV. (2007). Is the CVI an Acceptable Indicator of Content Validity? Appraisal and Recommendations. Res Nurs Health.

[19] Meliá JL. (1990). Construcción de la psicometría como ciência teórica y aplicada.

[20] Chan-Olmsted S, Wang R. (2020). Understanding podcast users: consumption motives and behaviors. New Media Soc..

[21] Barnes JH, Choby G, Smith AJ, Kiessling P, Marinelli JP, Bowe S (2020). Creation of a new educational podcast “headmirror’s ENT in a Nutshell”. Otolaryngol Head Neck Surg..

[22] Garcia EC, Costa IR, Oliveira RC, Silva CRL, Góis ARS, Abrão FMS. (2022). Social representations of adolescents about HIV/AIDS transmission in sexual relations: vulnerabilities and risks. Esc Anna Nery.

[23] Oliveira MPCA, Monteiro RJS, Belian RB, Lima LS, Gontijo DT. (2022). “Is deciding that you learn to decide”: validation of digital game on sexual and reproductive health in adolescence. Adolescenc Saúde.

[24] Malka R, Villwock J, Faucett E, Bowe S. (2021). Podcast-based learning in otolaryngology: availability, breadth, and comparison with other specialties. Laryngoscope.

[25] Katz M, Nandi N. (2020). Social media and medical education in the context of the COVID-19 pandemic: scoping review. JMIR Med Educ.

[26] Malecki S, Quinn KL, Zilbert N, Razak F, Ginsburg S, Verma AA (2019). Understanding the use and perceived impact of a medical podcast: qualitative study. JMIR Med Educ.

[27] Lameiras-Fernández M, Martínez-Román R, Carrera-Fernandez MV, Rodriguez-Castro Y. (2021). Sex education in the spotlight: what is working? Systematic review. Int J Environ Res Public Health.

[28] Perks LG, Turner JS, Tollison AC. (2019). Podcast uses and gratifications scale development. J Broadcast Electron Media.

[29] Souza V, Ramos KC, Matozinhos FP, Fonseca RMGS (2020). Validation of the Papo Reto game as a pedagogical device of adolescent in the context of sexuality. Rev Bras Enferm.

[30] Sousa MG, Oliveira EML, Coelho MMF, Miranda KCL, Henriques ACPT (2018). Validation of educational game for adolescents about the sexuality topic. Rev Fund Care Online.

